# US lesion visibility predicts clinically significant upgrade of prostate cancer by systematic biopsy

**DOI:** 10.1007/s00261-021-03389-x

**Published:** 2022-01-07

**Authors:** Nathan Velarde, Antonio C. Westphalen, Hao G. Nguyen, John Neuhaus, Katsuto Shinohara, Jeffry P. Simko, Peder E. Larson, Kirti Magudia

**Affiliations:** 1 Department of Radiology, University of California, San Francisco, San Francisco, CA USA; 2 Department of Urology, University of California, San Francisco, San Francisco, CA USA; 3 Department of Epidemiology & Biostatistics, University of California, San Francisco, San Francisco, CA USA; 4 Department of Pathology, University of California, San Francisco, San Francisco, CA USA; 5 Department of Radiology, Urology, and Radiation Oncology, University of Washington, Seattle, WA USA; 6 Department of Radiology, Duke University, Durham, NC USA

**Keywords:** Prostate neoplasms, Image-guided biopsy, Magnetic resonance imaging, Ultrasound

## Abstract

**Purpose:**

To identify predictors of when systematic biopsy leads to a higher overall prostate cancer grade compared to targeted biopsy.

**Methods and materials:**

918 consecutive patients who underwent prostate MRI followed by MRI/US fusion biopsy and systematic biopsies from January 2015 to November 2019 at a single academic medical center were retrospectively identified. The outcome was upgrade of PCa by systematic biopsy, defined as cases when systematic biopsy led to a Gleason Grade (GG) ≥ 2 and greater than the maximum GG detected by targeted biopsy. Generalized linear regression and conditional logistic regression were used to analyze predictors of upgrade.

**Results:**

At the gland level, the presence of an US-visible lesion was associated with decreased upgrade (OR 0.64, 95% CI 0.44–0.93, *p* = 0.02). At the sextant level, upgrade was more likely to occur through the biopsy of sextants with MRI-visible lesions (OR 2.58, 95% CI 1.87–3.63, *p* < 0.001), US-visible lesions (OR 1.83, 95% CI 1.14–2.93, *p* = 0.01), and ipsilateral lesions (OR 3.89, 95% CI 2.36–6.42, *p* < 0.001).

**Conclusion:**

Systematic biopsy is less valuable in patients with an US-visible lesion, and more likely to detect upgrades in sextants with imaging abnormalities. An approach that takes additional samples from regions with imaging abnormalities may provide analogous information to systematic biopsy.

## Introduction

The definitive diagnosis of prostate cancer (PCa) requires biopsy followed by histopathological analysis [1]. Historically, prostate biopsy samples have been obtained through multiple-core, systematic transrectal ultrasound-guided biopsy (i.e., systematic biopsy) [2]. Over the past several years, prostate MRI has become a routine component in the evaluation of suspected or known PCa [3–5]. Lesions visible on prostate MRI are more likely to represent clinically significant PCa (cs-PCa, Gleason score ≥ 3 + 4, GG ≥ 2), and the development of MRI/US fusion-guided biopsy (i.e., MRI-targeted biopsy) has enabled clinicians to accurately sample those lesions [5–10]. This approach may require fewer biopsies than systematic biopsy, and has been shown to diagnose more cs-PCa and less low-grade prostate cancer [4, 11–14].

Though MRI-targeted biopsy has the potential to improve diagnostic accuracy and decrease the number of unnecessary biopsies, concerns remain that cs-PCa may be missed when systematic biopsy is omitted. This concern has been confirmed by multiple recent studies that have shown that combined systematic and MRI-targeted biopsy maximizes diagnostic accuracy and minimizes missing cs-PCa [13, 15]. Though systematic biopsy continues to add value for some patients, the recommendation to perform systematic biopsy on all patients may lead to potentially unnecessary biopsies with possible complications as well as undue financial burden on individuals and the healthcare system.

An approach that uses pre-biopsy measures to predict when systematic biopsy would be of value could decrease the burden of unnecessary biopsies while maintaining diagnostic accuracy. One possible predictor could be the presence of lesions visible on transrectal ultrasound. Lesions visible on ultrasound but not MRI are also predictive of cs-PCa, and lesions visible on both are even more strongly predictive [7]. Additionally, PSA density has been shown to predict cs-PCa in MRI-negative patients and could be useful in identifying patients that would benefit from systematic biopsy [16].

We have assembled a large cohort of patients who underwent both MRI-targeted biopsy and systematic biopsy along with their relevant clinical data. This study aims to identify pre-biopsy measures under which systematic biopsy is unlikely to be of value.

## Methods

This study was approved by the UCSF institutional review board. All procedures were HIPAA compliant. Anonymized data available upon request.

### Cohort selection

1083 consecutive patients who underwent multiparametric prostate MRI followed by MRI/US fusion-guided biopsy and systematic biopsy from January 2016 to March 2019 at UCSF were retrospectively identified by queries of the electronic medical record (EMR) and radiology information systems. Pathology reports and demographic data were obtained from the EMR. Radiology reports were extracted from the radiology information systems and parsed for PSA density, gland volume, and PIRADS scores. 113 patients were excluded due to prior treatment, imaging artifacts, incomplete T2-weighted imaging (T2WI)/ apparent diffusion coefficient (ADC)/ diffusion weighted imaging (DWI) series, and incomplete biopsy data. For the 52 patients who were found to have undergone multiple prostate MRI exams followed by subsequent fusion and systematic biopsies, only the first pair was selected. The final cohort included 918 patients for analysis.

### MRI protocol

Multiparametric (MP) prostate MRI exams consisting of T2WI, DWI, and dynamic contrast-enhanced (DCE) imaging were acquired on multiple 3-Tesla Scanners (GE Healthcare, Waukesha, WI). Scans were interpreted as part of routine care by one of 16 subspecialized abdominal imaging radiologists with varying experience in prostate imaging. MRI-visible lesions were classified according to PI-RADS V2 [10]. A maximum of 4 MRI lesions were identified per patient and subsequently labeled using DynaCAD for Prostate (Invivo, Gainesville, FL, USA) prior to biopsy.

### Prostate biopsy protocol

All patients underwent biopsy at UCSF. Two urologists (HN and KS) performed all procedures in our cohort. The UroNav Fusion biopsy system (Philips) was used to superimpose axial T2WI images of the prostate over real time transrectal ultrasonographic scans. MRI/US fusion-guided biopsy was performed followed by biopsy of transrectal ultrasound (TRUS) visible lesions and finally TRUS-guided systematic biopsy. MRI identified lesions were sampled using an end fire biopsy device with transrectal ultrasonographic probe and guidance of the UroNav system. Discrete lesions visible on transrectal ultrasound as assessed by the urologist were sampled if present. Ultrasound lesions were present in 592 patients and were defined as hypoechoic lesions with vascularity. Well marginated lesions with smooth borders were considered more likely to be benign and were not biopsied. There was no size cutoff for ultrasound lesions. Depending on the size of the lesions and at the discretion of the urologist, one or two samples were taken from the center of the lesion and from its borders. TRUS-guided systematic biopsy was then performed. Samples were taken from the medial and lateral aspect of each sextant (right apex, right mid, right base, left apex, left mid, left base) with additional samples taken from the extended systematic sites (right anterior mid, left anterior mid) in 864 patients. A total of 12 or 14 cores were obtained depending on whether anterior biopsies were taken. All biopsies were performed by the same urologist in a single session.

### Histological analysis

A genitourinary pathologist (JS) with more than 20 years of experience with prostate cancer graded all specimens using Gleason scores and the 2014 International Society of Urological Pathology guidelines [1].

### Pathology data processing

Pathology reports were parsed for Gleason scores from biopsies taken from systematic biopsy sites, optional extended systematic biopsy sites, MRI lesions, and US lesions. The location of each lesion, the number of cores per biopsy, and the number of prostate cancer (PCa) positive cores per biopsy were also identified in each pathology report. MRI and US lesions were considered concordant if they occupied the same sextant.

### Statistical analysis

A mixed-effects logistic regression model was used to compare the rate of PCa detection between targeted biopsy and systematic biopsy at the gland level. The predictors were biopsy methods and the outcome was detection of clinically significant PCa (cs-PCa). A generalized linear regression was used to evaluate the association between PSA density and the detection of cs-PCa by systematic biopsy. A dichotomous PSA density was converted to a dichotomous variable (< 0.15, ≥ 0.15) to be subsequently used for all analyses.

The rate of clinically significant upgrade of prostate cancer, defined as cases in which cs-PCa is detected by systematic biopsy but not targeted biopsy, or in which the GG of cs-PCa detected by systematic biopsy is higher than that found by targeted biopsy, was first analyzed at the exam/patient level. GG 4 and 5 were grouped together for all analyses since they have similar treatment implications [17, 18]. Thus, an upgrade from GG 4 to GG 5 was not considered a clinically significant upgrade. A generalized linear regression was used to evaluate associations between potential predictors and clinically significant upgrade. Univariate analyses were conducted as well as a multivariate model adjusted for age and PSA density.

A conditional logistic regression was also used to compare the rate of PCa detection between sextants with different imaging characteristics. The unit of analysis was the individual sextant, the predictors were imaging characteristics, and the outcome was detection of cs-PCa.

At the sextant level, conditional logistic regression was used to evaluate associations between potential predictors and the detection of clinically significant prostate cancer by systematic biopsy of a given sextant. Univariate analyses were conducted using sextant level imaging characteristics including the presence of an MRI lesion, the presence of an US lesion, and the presence of an ipsilateral lesion. A multivariate analysis was conducted that included the presence of an MRI lesion, the presence of an US lesion, and the interaction term between these two predictors; this model was used to generate four odds ratios. Additional multivariate models included sextant level imaging characteristics and their interaction with PSA density.

All statistical analyses were performed by one of the authors (NV) in R statistical software (Version 1.2.5033, 2019, The R Foundation for Statistical Computing, Vienna, Austria, open source). All testing was two-tailed and p-values < 0.05 were considered statistically significant.

## Results

Patient characteristics and relevant imaging and biopsy data are described in Table 1.

**Table 1:** Demographic and clinical characteristics of included patients (*n* = 918)

Characteristic	All Patients (*n* = 918)	Known PCa (*n* = 493)	No known PCa (*n* = 425)
Age (years)	66.5 ± 7.3	66.3 ± 7.5	66.7 ± 6.9
Race or ethnicity*			
White	708 (77.1)	384 (77.9)	324 (76.2)
Black	33 (3.6)	16 (3.2)	17 (4.0)
Asian	57 (6.2)	34 (6.9)	23 (5.4)
Other	67 (7.3)	37 (7.5)	30 (7.1)
Unknown	53 (5.8)	22 (4.5)	31 (7.3)
PSA density (ng/ml^2^)	0.18 ± 0.21	0.16 ± 0.23	0.20 ± 0.18
Prostate Volume (cc)	54.2 ± 28.4	53.9 ± 27.8	54.5 ± 29.2
Number visible targets per prostate on MRI	1.48 ± 0.71	1.47 ± 0.72	1.50 ± 0.68
No. visible targets per prostate on US	0.76 ± 0.67	0.74 ± 0.68	0.78 ± 0.65
Maximum score on PI-RADS*			
1	0 (0)	0 (0)	0
2	2 (0.2)	1 (0.2)	1 (0.2)
3	195 (21.2)	111 (22.5)	84 (19.8)
4	419 (45.6)	232 (47.1)	187 (44.0)
5	302 (32.9)	149 (30.2)	153 (36.0)

Unless otherwise indicated, data are mean (standard deviation)

^*^Data are number (percentage)

### Cancer detection by biopsy method

**Fig. 1:**
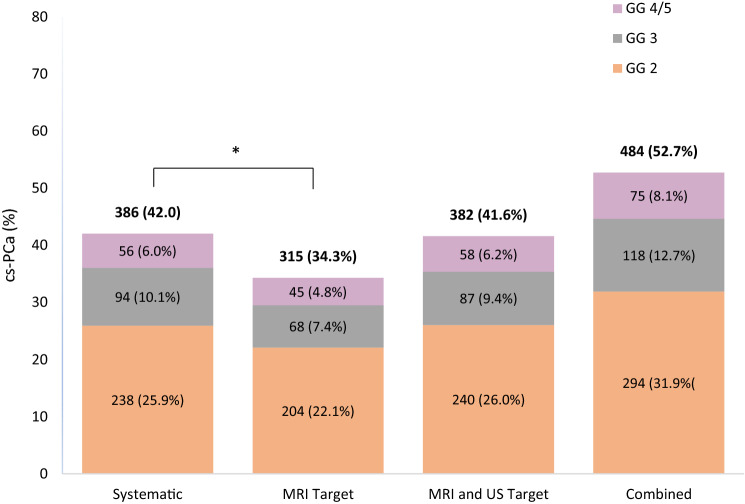
Rates of cs-PCa detection (ISUP Grade Group [GG] of 2 or greater) by systematic biopsy, MRI target biopsy, MRI and US target biopsy, and combined biopsy (systematic biopsy, MRI target biopsy, and US target biopsy; *n* = 918)

Of the 918 included patients, cs-PCa (GG ≥ 2) was detected in 386 (42.0%) of patients by systematic biopsy alone, 315 (34.3%) patients by MRI -targeted biopsy alone, 382 (41.6%) by either MRI-targeted or US-targeted biopsy (i.e., either targeted biopsy), and 484 (52.7%) by combined targeted and systematic biopsy (Fig. 1). Systematic biopsy alone detected more cs-PCa than MRI-targeted biopsy alone (*p* < 0.001). There was no difference between systematic biopsy and the combination of MRI-targeted and US-targeted biopsy in the rate of cs-PCa detection. Low-grade prostate cancer (GG 1) was detected in 371 (40.4%) patients by systematic biopsy alone, 235 (25.6%) patients by MRI-targeted biopsy alone, 238 (25.9%) patients by targeted biopsy, and 306 (33.3%) patients by targeted and systematic combined biopsy. Systematic biopsy alone detected more low-grade PCa than MRI and US-targeted biopsy (*p* < 0.001) or MRI-targeted biopsy alone (*p* < 0.001).

A total of 484 patients were diagnosed with cs-PCa by both systematic biopsy and MRI as well as US-targeted biopsy. MRI and US-targeted biopsy detected cs-PCa in 382 (79.0%) of those 484 patients, while systematic biopsy detected cs-PCa in 386 patients (79.8%, Table 2). Of the 918 patients included patients, systematic biopsy made clinically significant upgrades with GG ≥ 2 in 132 (14.4%) patients, with GG ≥ 3 in 55 patients (6.0%), and with GG ≥ 4 in 17 patients (1.8%, Table 2) compared to targeted biopsy. Conversely, targeted biopsy made clinically significant upgrades with GG ≥ 2 in 130 patients (14.2%), with GG ≥ 3 in 50 patients (5.4%), and with GG ≥ 4 in 19 patients (2.1%, Table 2) compared to systematic biopsy.

**Table 2:**
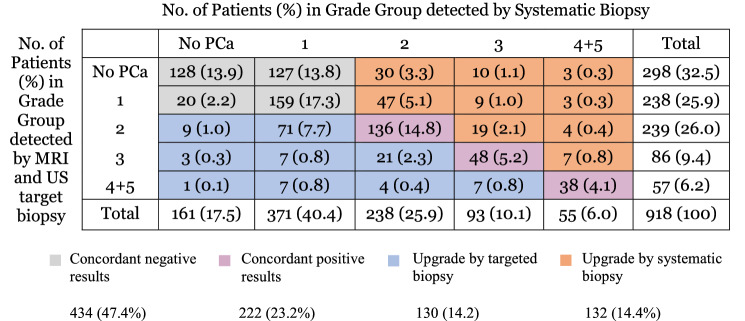
Cross-tabulation of systematic biopsy and targeted biopsy results with rates of concordance and upgrade

PSA density ≥ 0.15 ng/ml^2^ was associated with the detection of clinically significant prostate cancer by systematic biopsy (OR 2.66, 95% CI 2.03–3.49, *p* < 0.001).

### Predicting gland level upgrades by systematic biopsy

Upgrade by systematic biopsy occurred in 18.4% (60/326) of patients without US lesions and in 12.7% (75/592) of patients with US lesions. The presence of a visible US target was associated with a lower odds of upgrade by systematic biopsy [odds ratio (OR) 0.64, 95% confidence interval (CI) 0.44–0.93, *p* = 0.02, Table 3]. This association persisted in a model that included age and PSA density as predictors (OR 0.62, 95% CI 0.43–0.90, *p* = 0.01, Table 3). The presence of prior PCa, presence of concordant US/MRI lesions, maximum PIRADS score, PSA density, and age were not associated with clinically significant upgrade by systematic biopsy (Table 3).

**Table 3:** Odds ratios assessing the association of predictors with upgrade of clinically significant prostate cancer from mixed-effects logistic regression models at the gland level

Predictors	Category	Rate of upgrade	Odds ratio	*p*-value
Univariate model				
US target	Absent	60/326 (18.4%)	0.64 (95% CI 0.44–0.93)	0.02
	Present	75/592 (12.7%)		
Concordant US/MRI lesions	Absent	78/492 (15.9%)	0.82 (95% CI 0.57–1.19)	0.29
	Present	57/426 (13.4%)		
PSA density (ng/ml^2^)	< 0.15	68/503 (13.5%)	1.23 (95% CI 0.85–1.78)	0.26
	≥ 0.15	67/415 (16.1%)		
Known history of PCa	None	62/425 (14.6%)	Ref	ref
	Low-grade PCa	52/384 (13.5%)	0.92 (95% CI 0.62–1.36)	0.67
	High-grade PCa	21/100 (19.3%)	1.40 (95% CI 0.81–2.41)	0.23
Highest PIRADS score	2	0/2 (0.0%)	–	0.98
	3	23/195 (11.8%)	ref	ref
	4	74/419 (17.7%)	1.60 (95% CI 0.97–2.65)	0.07
	5	38/302 (12.6%)	1.08 (95% CI 0.62–1.87)	0.79
Age	–	–	1.01 (95% CI 0.99–1.04)	0.30
Gland volume	–	–	1.00 (95% CI 1.00–1.01)	0.24
Multivariate model				
Presence of US target	–	–	0.62 (95% CI 0.43–0.90)	0.01
PSA density	–	–	1.31 (95% CI 0.91–1.90)	0.15
Age	–	–	1.02 (95% CI 0.99–1.04)	0.25

### Sextant level cs-PCA detection

**Fig. 2:**
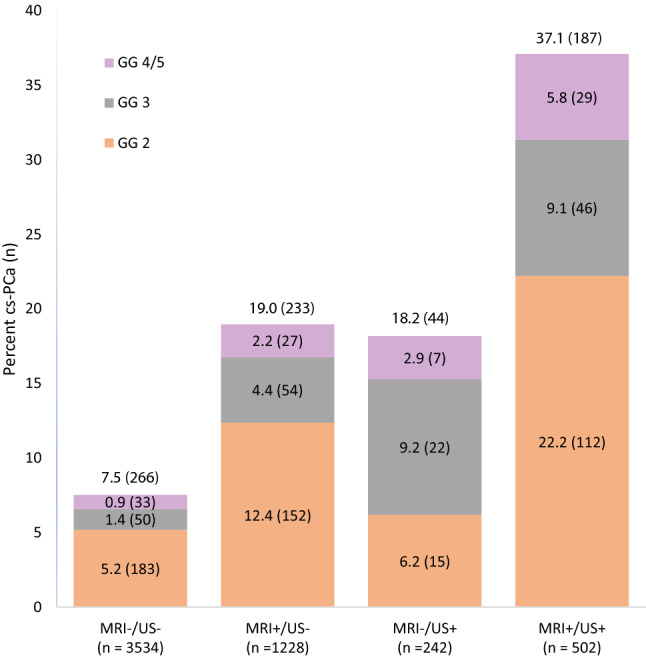
Sextant-level detection of cs-PCa by systematic biopsy in 5508 sextants based on the presence or absences of lesions visible on MRI and US

The 918 included patients comprised 5508 prostate gland sextants. Of these sextants, 3534 (64.2%) had no lesions visible on MRI or US, 1228 (22.3%) had lesions visible on MRI but not US, 242 (4.4%) had lesions visible on US but not MRI, and 504 (9.2%) had lesions visible on both MRI and US (Fig. 2). Cs-PCa was detected less often by systematic biopsy in sextants without MRI or US lesions [7.5% (266/3534)], and more often in sextants with lesions visible on MRI only [19.0% (233/1288)], US only [18.2% (44/242)], or both MRI and US [37.1% (187/504)] (Fig. 2). Furthermore, cs-PCa was detected least often [3.9% (59/1527)] in sextants without ipsilateral US or MRI lesion. Cs-PCa was significantly more likely to be detected on systematic biopsy in sextants with an MRI lesion (OR 4.15, 95% CI 3.37–5.10, *p* < 0.001), with an US lesion (OR 4.66, 95% CI 3.62–5.99, *p* < 0.001), and with an ipsilateral target (OR 9.03, 95% CI 6.41–12.72, *p* < 0.001).

### Predicting sextant level upgrades by systematic biopsy

Of the 5508 sextants from the 918 patients in our cohort sampled by systematic biopsy, 191 (3.5%) led to clinically significant upgrade by systematic biopsy that were not detected by MRI or US-targeted biopsy. The odds of an upgrade with systematic biopsy were significantly higher for sextants with an MRI target (OR 2.58, 95% CI 1.87–3.63, *p* < 0.001, Table 4). The presence of an MRI target was associated with upgrade in sextants without US lesions (OR 2.87, 95% CI 1.94–4.25, *p* < 0.001), but not in sextants with US lesions (OR 1.01, 95% CI 0.39–2.61, *p* = 0.98, Table 4). The odds of an upgrade with systematic biopsy were significantly higher for sextants with an US lesion (OR 1.83, 95% CI 1.14–2.93, *p* = 0.01. Table 4). The presence of an US target was associated with upgrade in sextants without MRI lesions (OR 2.45, 95% CI 1.08–5.58, *p* = 0.03), but not in sextants with MRI lesions (OR 0.86, 95% CI 0.46–1.62, *p* = 0.65, Table 4). Clinically significant upgrades were also statistically more likely to be made in sextants with ipsilateral lesions compared to those without (OR 3.89, 95% CI 2.36–6.42, *p* < 0.001, Table 4). The association of MRI lesions, US lesions, and ipsilateral lesions did not differ by PSA density.

**Table 4:** Odds ratios assessing the association of predictors with upgrade of clinically significant prostate cancer from conditional logistic regression models at the sextant level

Predictors	Category	Rate of upgrade	Odds ratio	*p*-value
Univariate model				
MRI target	Absent	100/3776 (2.65%)	2.58 (95% CI 1.87–3.63)	< 0.001
	Present	91/1732 (5.25%)		
US target	Absent	158/4762 (3.32%)	1.83 (95% CI 1.14–2.93)	0.01
	Present	33/746 (4.42%)		
Ipsilateral target	Absent	23/1527 (1.51%)	3.89 (95% CI 2.36–6.42)	< 0.001
	Present	168/3981 (4.22%)		
Multivariate model 1				
MRI target (in US- sextants)	Absent	90/3534 (2.55%)	2.87 (95% CI 1.94–4.25)	< 0.001
	Present	68/1228 (5.54%)		
MRI target (in US + sextants)	Absent	10/242 (4.13%)	1.01 (95% CI 0.39–2.61)	0.98
	Present	23/504 (4.56%)		
US target (in MRI- sextants)	Absent	90/3534 (2.55%)	2.45 (95% CI 1.08–5.58)	0.03
	Present	10/242 (4.13%)		
US target (in MRI + sextants)	Absent	68/1228 (5.54%)	0.86 (95% CI 0.46–1.62)	0.65
	Present	23/504 (4.56%)		
Multivariate model 2				
MRI target	–	–	2.14 (95% CI 1.32–3.45)	0.002
MRI target:PSA density	–	–	1.47 (95% CI 0.74–2.94)	0.27
Multivariate model 3				
US target	–	–	1.77 (95% CI 0.87–3.59)	0.12
US target:PSA density	–	–	1.06 (95% CI 0.41–2.75)	0.90
Multivariate model 4				
Ipsilateral lesion	–	–	2.52 (95% CI 1.32–4.82)	0.005
Ipsilateral lesion:PSA density	–	–	2.63 (95% CI 0.94–7.38)	0.07

## Discussion

Although a combined approach using both systematic and targeted biopsy methods has the potential to minimize diagnostic uncertainty, identifying scenarios in which systematic biopsy could be avoided while maintaining diagnostic accuracy could reduce the burden of unnecessary biopsies. To our knowledge, this is the first study to identify predictors that indicate when systematic biopsy adds value to targeted biopsy. In summary, we found that systematic biopsy is less likely to make a clinically significant upgrade of prostate cancer in patients with a visible US lesion, and that upgrades are more likely to occur through the systematic biopsy of sextants with MRI or US lesions.

In our gland wide analysis, we found that systematic biopsy was significantly less likely to make a clinically significant upgrade of prostate cancer in patients with a lesion visible on transrectal ultrasound. Many lesions visible on US may be the same lesions visible on MRI. When clinicians visualize these lesions on US, it may prompt them to take additional biopsies of the relevant sextants. The increased number of biopsies taken from suspicious regions of the prostate in these scenarios could increase the accuracy of targeted biopsy, and decrease the chance that systematic biopsy would lead to a clinically significant upgrade. On the other hand, it is possible that when clinicians see a lesion on US, they sample less suspicious areas with systematic biopsy and that this approach leads to a decreased rate of upgrade. While prostate MRI has become part of routine clinical practice in the evaluation and management of prostate cancer, our results show that transrectal ultrasound continues to add value in prostate cancer diagnosis.

Our sextant-based analysis shows that when systematic biopsy made clinically significant upgrades, it was more likely to occur through the biopsy of sextants with a lesion visible on MRI or US. Upgrades were also significantly less likely to occur in sextants without ipsilateral MRI or US lesions. These results suggest that upgrades primarily occur when the region of an MRI or US lesion with the highest GG is missed by targeted biopsy, but presumably sampled by systematic biopsy. While the presence of US lesions decreases the overall likelihood of upgrade by systematic biopsy at the gland level, upgrades by systematic biopsy are most likely to occur through the biopsy of sextants that are suspicious on MRI and US.

Our sextant-based analysis also found that cs-PCa was more likely to be detected by systematic biopsy in sextants with MRI lesions or US lesions and was less likely to be detected in sextants without ipsilateral lesions. These results may be particularly useful when considering the use of focal therapy to treat cs-PCa [19, 20]. The knowledge that cs-PCa is less likely to be detected in sextants without ipsilateral lesions may give clinicians more confidence to treat MRI and US lesions focally with decreased concern of missing prostate cancer in regions of the prostate without imaging abnormalities.

In 2020, Pagniez et al. showed that PSA density predicts the detection of cs-PCa by systematic biopsy in MRI-negative patients [16]. Further, PSA density was also shown to be an independent predictor of cs-PCa in men with visible lesions on MRI [21, 22]. While our results found that PSA density predicts the detection of cs-PCa by systematic biopsy, PSA density did not predict cancer upgrade on systematic biopsy. This may be because systematic biopsy tends to identify invisible lesions that are likely low volume with little impact on PSA density compared to targeted lesions that represent a large volume of disease with higher impact on PSA density.

The generalizability of this study may be limited as the image interpretation, prostate biopsies, and histopathological analyses were all performed at a single academic medical center by experts in prostate cancer diagnosis. Differences in biopsy procedures including the biopsy of transrectal US-visible lesions, routine inclusion of anterior prostate biopsy sites, and altering systematic biopsy sites given target lesions may limit the generalizability of this study [4, 12, 13]. Our results, however, suggest that sampling transrectal US-visible lesions improves PCa characterization. It is also possible that upgrades from GG 2 (Gleason score 3 + 4) to GG 3 (Gleason score 4 + 3) may be due to sampling more Gleason Score 4 than 3 in a biopsy than representative of the surrounding tissue. Given that many PCa patients do not proceed to surgery, we were not able to correlate our systematic and/or targeted biopsy results to whole mount histopathological analysis. Lastly, a urologist sampled MRI targets, identified and sampled US targets, and then obtained systematic biopsies in a single session. Knowledge of the location of MRI lesions almost certainly influenced the detection of transrectal ultrasound lesions and how systematic biopsies were performed.

## Conclusion

Systematic biopsy is less likely to detect clinically significant upgrades in patients with US-visible lesions and suggests a clinical scenario in which systematic biopsy may provide less value. Upgrades made by systematic biopsy are more likely to occur in sextants with imaging abnormalities and less likely to occur on sides of the prostate without ipsilateral lesions. This information suggests that an approach that takes additional samples from sextants containing or near to visible MRI or US lesions may provide the same information that would be obtained through systematic biopsy; this requires validation at other institutions and should be studied prospectively.

## Data Availability

Data are available upon request.

## Code availability

Code is available upon request.
